# *Porphyromonas gingivalis* outer membrane vesicles inhibit the invasion of *Fusobacterium nucleatum* into oral epithelial cells by downregulating FadA and FomA

**DOI:** 10.1002/JPER.21-0144

**Published:** 2021-10-05

**Authors:** Zhiying Zhang, Sai Liu, Shuwei Zhang, Yuchao Li, Xiaoting Shi, Dongjuan Liu, Yaping Pan

**Affiliations:** ^1^ Department of Periodontics, Liaoning Provincial Key Laboratory of Oral Diseases, School and Hospital of Stomatology China Medical University Shenyang China; ^2^ Department of Dental Materials, Liaoning Provincial Key Laboratory of Oral Diseases, School and Hospital of Stomatology China Medical University Shenyang China; ^3^ Department of Emergency and Oral Medicine, Liaoning Provincial Key Laboratory of Oral Diseases, School and Hospital of Stomatology China Medical University Shenyang China

**Keywords:** *Fusobacterium nucleatum*, invasion ability, oral epithelial cells, outer membrane vesicles, *Porphyromonas gingivalis*

## Abstract

**Background:**

*Porphyromonas gingivalis* (*P. gingivalis*) and *Fusobacterium nucleatum* (*F. nucleatum*) participate in the formation and progression of periodontitis. They can exert virulence by invading into host cells, but the interaction between them and their specific mechanisms remain unclear. The purpose of this study was to study the effect of *P. gingivalis* outer membrane vesicles (OMVs) on the ability of *F. nucleatum* to invade oral epithelial cells, and the reasons for the influence.

**Methods:**

The invasion abilities of the two bacteria were detected separately after mixed infection of *P. gingivalis* and *F. nucleatum*. Next, *P. gingivalis* OMVs were extracted with the kit, and their influence on the invasion ability of *F. nucleatum* was tested. The effects of *P. gingivalis* OMVs on *F. nucleatum* were evaluated by assessment of bacterial morphology, growth curves, auto‐aggregation morphology, and the expression of adhesion‐related proteins FadA and FomA.

**Results:**

Our results showed that *P. gingivalis* inhibited the invasion of *F. nucleatum* into oral epithelial cells but *F. nucleatum* promoted the invasion of *P. gingivalis*. In subsequent experiments, we extracted *P. gingivalis* OMVs successfully and revealed that proteases in *P. gingivalis* OMVs inhibited the invasion of *F. nucleatum* into oral epithelial cells. Furthermore, *P. gingivalis* OMVs did not affect the morphology and proliferation of *F. nucleatum*, but proteases inside decreased the auto‐aggregation of *F. nucleatum*. Additionally, proteases in *P. gingivalis* OMVs reduced the expression levels of *F. nucleatum* surface adhesion‐related proteins FadA and FomA.

**Conclusion:**

Our study demonstrated that proteases in *P. gingivalis* OMVs inhibited the invasion of *F. nucleatum* into oral epithelial cells by downregulating FadA and FomA.

## 1 INTRODUCTION

Periodontitis can cause the destruction of periodontal supporting tissues and is closely related to a variety of systemic diseases.^1^ As important pathogenic bacteria, *Porphyromonas gingivalis* (*P. gingivalis*) and *Fusobacterium nucleatum* (*F. nucleatum*) play vital roles in the occurrence and development of periodontitis.^2^

*P. gingivalis* is a gram‐negative anaerobic black‐pigmented bacteria that forms a “red complex” with *Treponema denticola* and *Tannerella forsythia*, and is one of the major pathogens of periodontitis.^3^ *F. nucleatum* is a common opportunistic pathogen of the oral microflora, which links early and late colonizers like a bridge, it functions in the process of plaque biofilm formation, bacterial colonization, and mixed infection.^4^ Both *P. gingivalis* and *F. nucleatum* can bind to corresponding cell ligands through specific adhesin to activate multiple signaling pathways, and finally internalize in the host cells.^5^, ^6^ Numerous studies have demonstrated that *P. gingivalis* could invade into human gingival epithelial cells^5^, gingival fibroblasts^7^, vascular endothelial cells^8^ and periodontal ligament stem cells,^9^ and *F. nucleatum* could invade into oral, colonic, placental epithelial cells, immune cells, keratinocytes and so on.^10^ The internalized bacteria affected the synthesis and secretion of certain cytokines, regulated cell proliferation,^11^, ^12^ apoptosis^13^, ^14^ and other biological behaviors, leading to epithelial cell dysfunction and the destruction of periodontal tissue.

The interaction between different microbial populations affects the occurrence and development of host diseases.^15^ Therefore, it is necessary to explore the characteristics of different pathogenic bacteria and the virulence mechanism between their interactions. Studies have confirmed that *P. gingivalis* can inhibit the invasion of *F. nucleatum* into gingival epithelial cells through a gingipains‐dependent mechanism. It is worth noting that previous experiments could not prove the effect of the interaction between *P. gingivalis* and *F. nucleatum* during mixed infection, and the specific mechanism that caused the change of invasion ability is still unclear.^16^

During the growth of *P. gingivalis*, double‐layer spherical membrane‐like vesicles called outer membrane vesicles (OMVs) are continuously secreted from the cell surface.^17^ Because of the protection of the vesicle membrane structure, the highly concentrated pathogenic factors can avoid degradation and achieve long‐distance delivery, making *P. gingivalis* OMVs exhibit stronger toxicity than the parent bacteria.^18^, ^19^ Therefore, OMVs may represent *P. gingivalis* to interact with other oral bacteria^20^ and affect the invasion of *F. nucleatum*.

In this research, we uncovered that *P. gingivalis* OMVs decreased the auto‐aggregation of *F. nucleatum*, and downregulated the expression of *F. nucleatum* surface adhesion‐related proteins FadA and FomA, thereby inhibiting the invasion of *F. nucleatum* into oral epithelial cells. It provides a new evidence for the interaction between different bacteria and enriches the virulence mechanism of *P. gingivalis* as one of major oral pathogens.

## 2 MATERIALS AND METHODS

### 2.1 Bacteria and cell culture

*P. gingivalis* ATCC 33277 and *F. nucleatum* ATCC 25586 were grown anaerobically (10% CO_2_, 10% H_2_, 80% N_2_) on brain heart infusion (BHI) agar plates supplemented with 5% defibrinated sheep blood, 5 μg/mL hemin, 0.1% vitamin K1, and 0.5 mg/mL yeast extract. The bacteria on the agar plate were scraped into BHI liquid medium until logarithmic growth phase before the experiment operation. Human immortalized oral epithelial cells (HIOECs), which had been obtained and immortalized from the normal oral mucosa of patients undergoing cleft palate or lip reconstruction surgery as previously described,^21^ were cultured in defined keratinocyte serum‐free medium (Gibco, Thermo Fisher Scientific Inc., Waltham, MA, USA.) with 25 μg/mL bovine pituitary extract and 0.2 ng/mL epidermal augmentum factor at 37°C with 5% CO_2_ in a humidified atmosphere. The cell line was generously provided by Key Laboratory of Shanghai Oral Medicine, Shanghai Jiao Tong University. Bacteria were added to the cells at a multiplicity of infection (MOI) of 100 in both single and mixed infection.

### 2.2 Preparation of *P. gingivalis* OMVs

OMVs were isolated from *P. gingivalis* ATCC 33277 by exobacteria OMV isolation kit for gram negative bacteria (System Biosciences Co., Palo Alto, CA, USA). Relevant reagents detected by test paper are neutral liquids. Thirty milliliter of bacterial liquid was collected and centrifuged twice at 5000 × *g* for 20 minutes at 4℃. The supernatant was collected and passed through 0.45‐μm‐pore‐size and 0.22‐μm‐pore‐size filters successively, and incubated in a suspension instrument for 30 minutes at 4℃ after being poured into the separation column with binding resin. After washing the column three times with binding buffer, the *P. gingivalis* OMVs were eluted and collected with the OMV elution buffer, and stored at –80℃. The concentration was determined by BCA protein analysis kit (BestBio. Co., China) before use.^22^ The heat‐inactivated OMV group and protease inhibitor phenylmethylsulfonyl fluoride (PMSF, Boster Biological Technology, Pleasanton, CA, USA) group were added in subsequent experiments. The heat‐inactivated condition of *P. gingivalis* OMVs in the heat‐inactivated group was 100°C for 30 minutes.^23^ In the PMSF group, 1 μg/mL *P. gingivalis* OMVs were incubated with 10 mM PMSF in advance at 37°C for 30 minutes to inhibit the activity of proteases.^24^ In addition, 10 mM PMSF adds to the *F. nucleatum* solution directly was included as a negative control (PMSF‐NC) of the protease inhibitor in the same experiment. According to references and the results of the preliminary experiment (see Supplementary Figure 1 in online *Journal of Periodontology*), every 10^7^ CFU/mL *F. nucleatum* was treated with 1 μg/mL *P. gingivalis* OMVs in advance for 6 hours,^25^, ^26^, ^27^ and then the follow‐up experiment was carried out.

### 2.3 Identification and observation of *P. gingivalis* OMVs

The extracted *P. gingivalis* OMVs were collected in tubes, and the diameter and particle number were measured using nanoparticle tracking analysis (NTA) (Particle Metrix Co., Germany). In addition, *P. gingivalis* OMVs were fixed in 2.5% glutaraldehyde, and then dehydrated in graded acetone and embedded. The sample was cut into thin sections, and the morphology was observed with a transmission electron microscope (TEM) (HT7700, Hitachi, Japan).

### 2.4 Flow cytometry analysis of the invasion ability of *P. gingivalis* and *F. nucleatum*

Flow cytometry was used to evaluate bacterial invasion, as previously reported.^16^, ^28^ In the experiment of mixed infection, the concentration of bacteria to be tested was adjusted to 10^9^CFU/mL and labeled with 10 μM 5‐(and 6)‐carboxyfluorescein diacetate succinimidyl ester (CFSE) (Invitrogen, Thermo Fisher Scientific Inc., Waltham, MA, USA). Afterwards, the two bacteria were mixed equally and pre‐incubated for 15 minutes, and incubated together with HIOECs for 4 hours when cells were grown to near‐confluence. In experiments involving *P. gingivalis* OMVs, *F. nucleatum* was treated with *P. gingivalis* OMVs in advance, and *F. nucleatum* was labeled with CFSE and incubated with HIOECs then. Following incubation, the cells were washed four times with sterile PBS, and 400 μg/mL trypan blue (Gibco, Thermo Fisher Scientific Inc., Waltham, MA, USA) was added to quench the fluorescent labeling of extracellular bacteria. HIOECs were detached with trypsin (Gibco, Thermo Fisher Scientific Inc., Waltham, MA, USA) and collected, and the fluorescence intensity of the bacteria in the cells was analyzed by flow cytometry (FACS, BD, Franklin Lakes, NJ, USA). After gating the HIOECs based on the forward and side scatter of uninfected cells, the fluorescence intensity of 10,000 cells in each sample was measured in the FITC‐A fluorescence channel for detecting CFSE fluorescence. Compared with uninfected cells, cells containing the bacteria to be tested showed significantly increased fluorescence intensity.

### 2.5 Confocal microscopy observation of invasion ability of *F. nucleatum*

After HIOECs was cultured on confocal dishes to near‐confluence, the *F. nucleatum* which have been treated with *P. gingivalis* OMVs was added. The medium was discarded after 4 hours and the samples were fixed with 4% paraformaldehyde for 10 minutes. After washing with PBS and blocking with 1% BSA for 1 hour, the sample was incubated with primary antibody overnight at 4°C, and then incubated with Alexa Fluor 594‐conjugated secondary antibody (1:200 dilution; Proteintech Group, Chicago, IL, USA.) at 37°C for 2 hours. Next, the sample was treated with 0.5% Triton X‐100 and blocked with 3% BSA again. Following overnight incubation with the primary antibody, the sample was incubated with Alexa Fluor 488‐conjugated secondary antibody (1:200 dilution; Proteintech Group, Chicago, IL, USA.) at 37°C for 1 hour. Nucleus of HIOECs was stained with 4′‐6‐diamidino‐2‐phenylindole (DAPI) (Beyotime Biotech. Co., China) and mounted with anti‐fluorescence quencher for observation. Laser confocal microscopy imaging system (Leica TCS SP5, Wetzlar, Germany) was used to image and photograph of the sample. The primary antibody involved in this experiment is a polyclonal antibody against *F. nucleatum* obtained after immunizing New Zealand white rabbits with inactivated *F. nucleatum* for 7 weeks.

### 2.6 Morphological observation of *F. nucleatum*

*F. nucleatum* on the agar plate were scraped into BHI liquid medium. The bacterial solution in the logarithmic growth phase was transferred to a well plate containing sterile coverslips coated by poly‐d‐lysine (Meilunbio, Da Lian, China), and *P. gingivalis* OMVs were added to the wells. The sample was fixed in 2.5% glutaraldehyde overnight after 6 hours of treatment and washed with 0.1 M PBS. Next, the sample was added with 1% osmium acid for 1 hour, then washed twice again and dehydrated with gradient ethanol (30%‐50%‐70%‐100%). After critical point drying (Leica EM CPD200, Wetzlar, Germany), the sample was coated with Pt (JEC‐300FC automatic ion sputtering instrument, JEOL, Japan) and finally observed under scanning electron microscope (SEM) (JSM‐IT200, JEOL, Japan).

### 2.7 Growth curve analysis and auto‐aggregation observation of *F. nucleatum*

After treatment with *P. gingivalis* OMVs, the bacterial solution of *F. nucleatum* was added in a 96‐well plate. The growth curves of *F. nucleatum* was monitored by measuring the absorbance value at 600 nm at 2, 4, 6, and 8 hours with a microplate reader (Tecan, Groedig, Austria). Furthermore, auto‐aggregation of bacteria was observed as described previously^29^, the auto‐aggregation of *F. nucleatum* was photographed at 6 hours under different magnification of optical microscopes (Nikon 80i, Tokyo, Japan).

### 2.8 Western blot analysis of *F. nucleatum* surface adhesion‐related proteins FadA and FomA

*F. nucleatum* total protein was extracted alone or in the presence of *P. gingivalis* OMVs with the total bacterial protein extraction kit (BestBio. Co., China), and was quantified with BCA protein analysis kit (BestBio. Co., China). Equal amounts of protein were loaded onto 12% SDS/PAGE gels and transferred to PVDF membranes. After blocking the membranes with TBST solution containing 5% bovine serum albumin, the membranes were incubated with the primary antibody at 4°C overnight and then incubated with the fluorescent secondary antibody (Proteintech, USA). Finally, images were acquired by Odyssey CLX (LICOR, Lincoln, NE, USA) and the protein band was analyzed by Image J software (NIH). The primary antibodies involved in this experiment include anti‐FadA, anti‐FomA (1:1000) (According to the gene sequences of FadA and FomA [GeneBank: DQ012971.1 and GenBank: X72582.1], the plasmids were constructed and transferred into *Escherichia coli* to secrete the proteins. The purified proteins were made into antigens, and New Zealand white rabbits were immunized with the antigens for 7 weeks to obtain antibodies of FadA and FomA; the evaluation of their specificity is shown in Supplementary Figure 2.) and anti‐DnaK (as internal control^30^, ^31^) (1:1000; Abcam, Cambridge, MA, USA).

### 2.9 Statistical analysis

After all data were tested independently for at least three times under each condition, SPSS 26 software (SPSS, Inc., Chicago, IL, USA) was used for statistical analysis. The Student's *t*‐test was used to compare the differences between the two groups. When the *P* value was less than 0.05, the difference was considered to be statistically significant.

## 3 RESULTS

### 3.1 *P. gingivalis* inhibited the invasion of *F. nucleatum* into HIOECs, but *F. nucleatum* promoted the invasion of *P. gingivalis* into HIOECs

**FIGURE 1:**
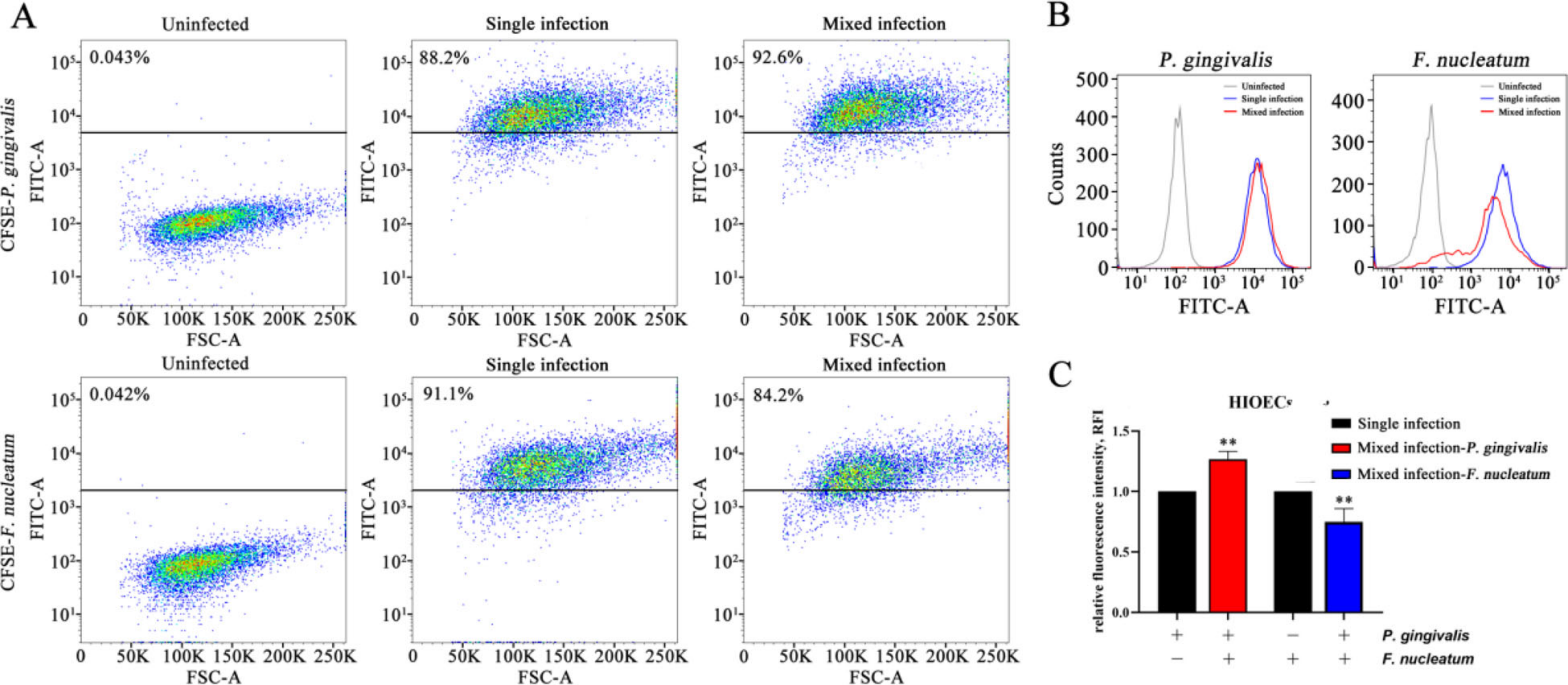
*P. gingivalis* inhibited the invasion of *F. nucleatum* into HIOECs, but *F. nucleatum* promoted the invasion of *P. gingivalis* into HIOECs. (**A**) Representative flow cytometry scatter plots of HIOECs infected with CFSE‐labeled bacteria. (**B**) Representative flow peak plots of HIOECs infected with CFSE‐labeled bacteria. (**C**) The relative fluorescence intensity (RFI) of the cells was analyzed by calculating the data. The data are presented as the mean ± SD obtained from three independent experiments (*n* = 3). *P < 0.05, **P < 0.01 versus the control cells (Student's *t* test)

In order to explore the impact of mixed infection on the invasion ability of *P. gingivalis* and *F. nucleatum*, the bacteria to be tested were labeled with CFSE and then infected with HIOECs in the presence or absence of the other bacteria. We used flow cytometry to detect the invasion ability of bacteria, and representative images of scatter plots and peak plots were shown in Figure 1A,B. HIOECs containing CFSE‐labeled *P. gingivalis* increased from 88.2% to 92.6% and the curve moved slightly to the right, whereas HIOECs containing CFSE‐labeled *F. nucleatum* decreased from 91.1% to 84.2% and the curve moved to the left after mixed infection. Compared with the single infection group, the relative fluorescence intensity of *P. gingivalis* of the mixed infection group increased, and the relative fluorescence intensity of *F. nucleatum* in the HIOECs decreased (Figure 1C). These results indicated that *P. gingivalis* inhibited the invasion of *F. nucleatum* into oral epithelial cells, but *F. nucleatum* promoted the invasion of *P. gingivalis* into oral epithelial cells.

### 3.2 *P. gingivalis* OMVs inhibited the invasion of *F. nucleatum* into HIOECs

**FIGURE 2:**
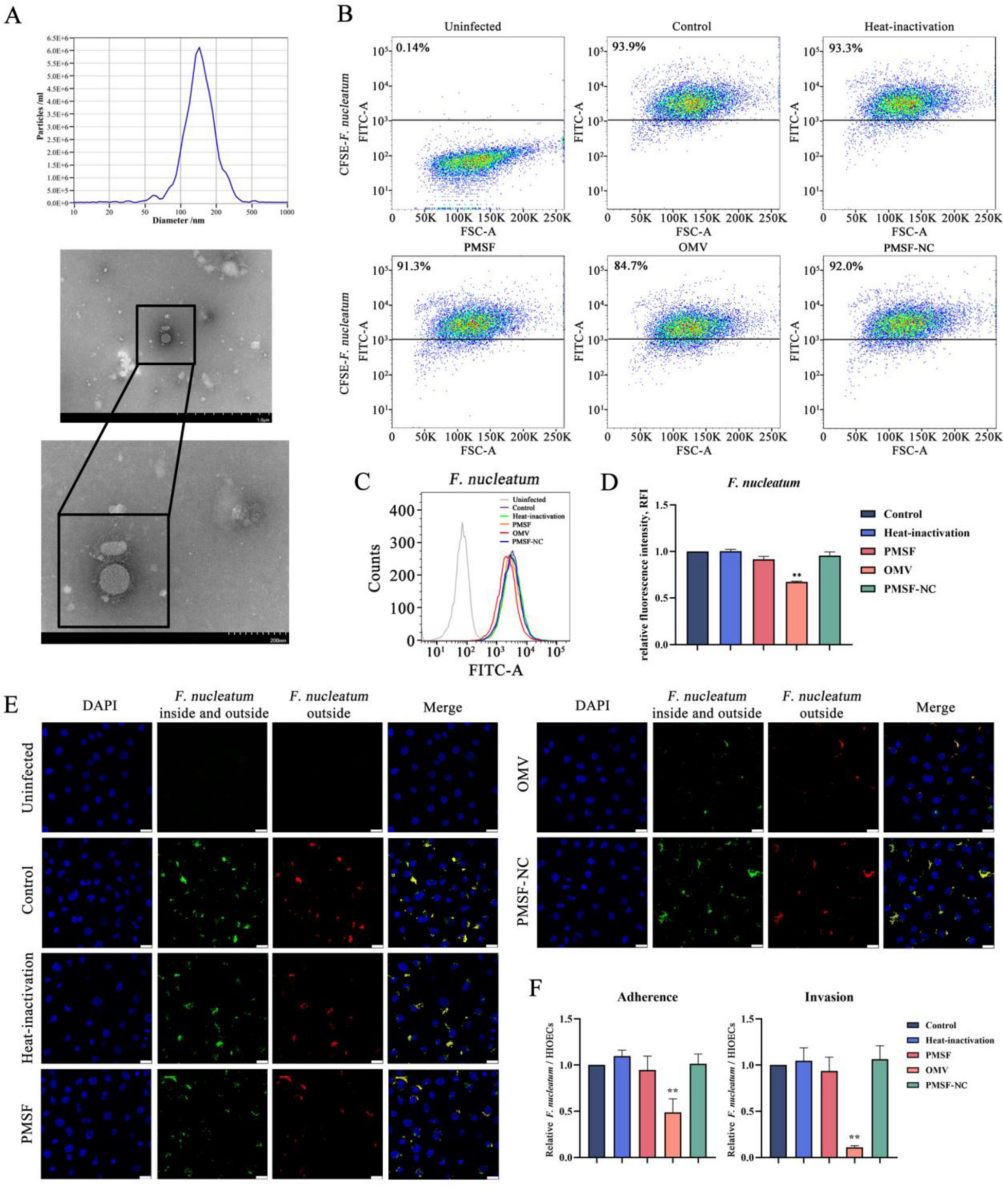
*P. gingivalis* OMVs inhibited the invasion of *F. nucleatum* into oral epithelial cells, PMSF inhibited this effect of *P. gingivalis* OMVs. (**A**) NTA of number and size distribution in *P. gingivalis* OMVs, and TEM images of purified *P. gingivalis* OMVs. Scale bars = 1.0 μm and 200 nm. (**B**) Following treatment with *P. gingivalis* OMVs for 6 hours, *F. nucleatum* was labeled with CFSE and used to infect HIOECs for 4 hours, then the representative flow cytometry scatter plots were acquired. In addition, the heat‐inactivation group of OMVs treated with heat inactivation at 100°C for 30 minutes, the PMSF group of OMVs treated with PMSF at 37°C for 30 minutes, and the PMSF‐NC group where PMSF was directly added to *F. nucleatum* were also added. (**C**) Representative flow peak plots were acquired in the same way as B. (**D**) The relative fluorescence intensity (RFI) of HIOECs infected with CFSE‐labeled *F. nucleatum* was analyzed by calculating the data. (**E**‐**F**) Representative confocal microscopy images of and relative quantitative analysis of adhesion and invasion of *F. nucleatum* were acquired. *F. nucleatum* internalized into HIOECs was green and bacteria adhered to the cell surface was yellow in merge part. Scale bar = 25 μm. The data are presented as the mean ± SD obtained from three independent experiments (*n* = 3). *P < 0.05, **P < 0.01 vs. the control cells (Student's *t* test). PMSF: phenylmethylsulfonyl fluoride; PMSF‐NC: phenylmethylsulfonyl fluoride negative control; NTA: nanoparticle tracking analysis; TEM: transmission electron microscope

*P. gingivalis* OMVs were extracted from the *P. gingivalis* by exobacteria OMV isolation kit for gram negative bacteria, and identified through NTA and TEM. As shown in Figure 2A, a typical round vesicle‐like morphology was observed with an average diameter of about 146.2 nm. It suggested that we extracted *P. gingivalis* OMVs successfully and laid the foundation for follow‐up research. We used flow cytometry and laser confocal scanning microscopy to explore the effect of *P. gingivalis* OMVs on the ability of *F. nucleatum* to invade HIOECs. *F. nucleatum* treated with *P. gingivalis* OMVs was used to infect HIOECs at a MOI of 100:1 for 4 hours for subsequent detection. As representative images of scatter plots and peak plots were shown in Figure 2B,C, the percentage of *F. nucleatum* in HIOECs decreased from 93.9% to 84.7% and the curve moved to the left after *P. gingivalis* OMVs treatment, whereas there was no significant difference in the percentage of *F. nucleatum* treated with heat‐inactivated or PMSF pre‐incubated OMVs. There was also no difference in the PMSF‐NC group, which ruled out the direct impact of PMSF on *F. nucleatum*. As shown in Figure 2D, the relative fluorescence intensity of *F. nucleatum* treated with *P. gingivalis* OMVs in HIOECs was significantly reduced compared with the control group, whereas the *F. nucleatum* treated with heat‐inactivated OMVs, PMSF pre‐incubated OMVs and PMSF did not change. Then we obtained more intuitive images through fluorescent staining. As shown in Figure 2E, the green fluorescence was the *F. nucleatum* inside and outside the cells, which represented the bacteria that adhere and invade. The red fluorescence was the *F. nucleatum* outside the cells, which represented attached bacteria. In the merged image, yellow fluorescence represented bacteria adhering to the cell surface, and green fluorescence represented internalized bacteria. As shown in Figure 2F, the adhesion and invasion of *F. nucleatum* in the fluorescence image were analyzed. It can be seen that the adhesion and invasion ratios of *F. nucleatum* treated with OMVs were reduced, and there was no difference in other groups, which were consistent with the results of flow cytometry. These results indicated that *P. gingivalis* OMVs inhibited the invasion of *F. nucleatum* into HIOECs.

### 3.3 *P. gingivalis* OMVs decreased the auto‐aggregation of *F. nucleatum* and downregulated the expression of *F. nucleatum* surface adhesion‐related proteins FadA and FomA

**FIGURE 3:**
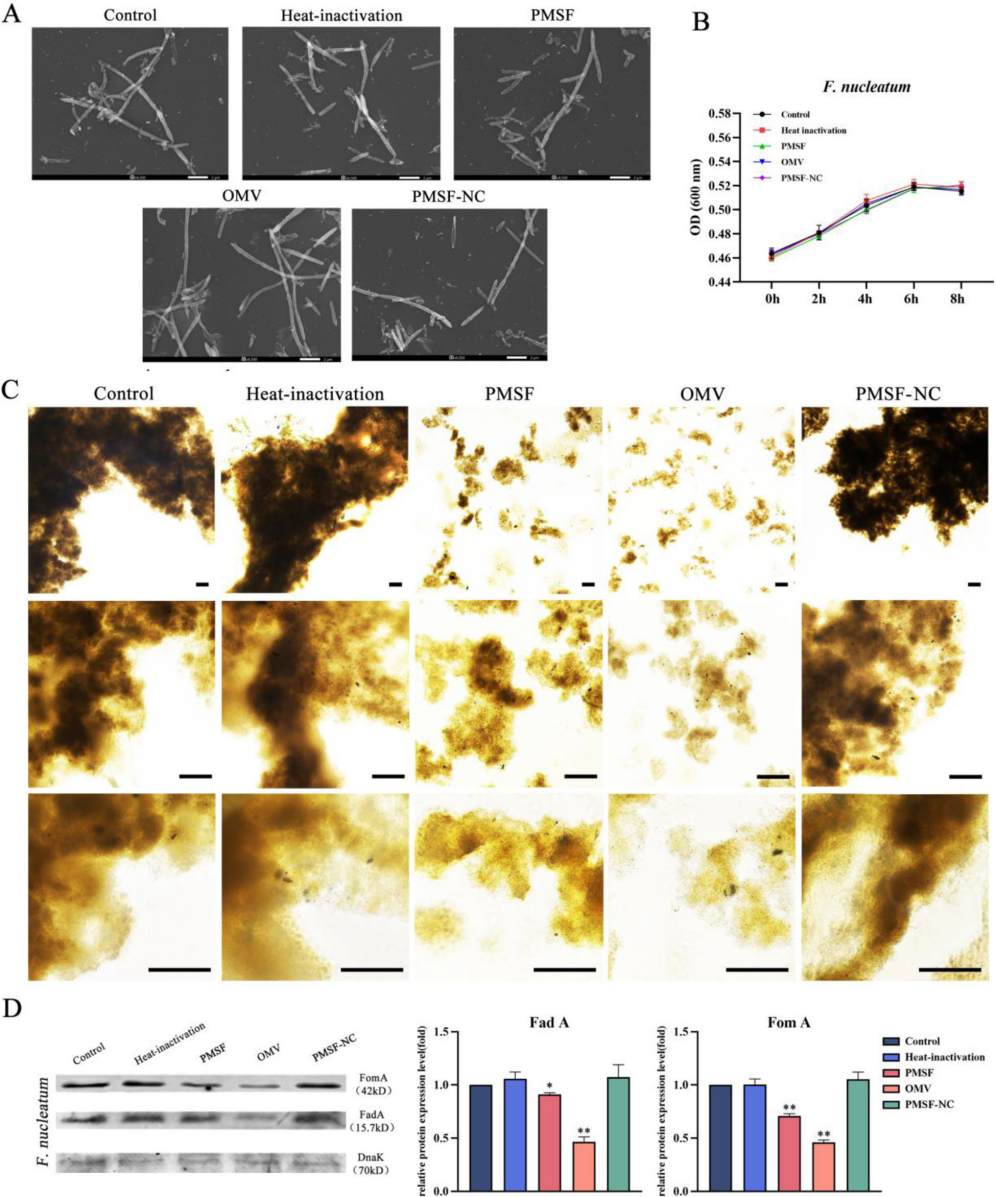
*P. gingivalis* OMVs decreased the auto‐aggregation of *F. nucleatum* and downregulated the adhesion‐related proteins FadA and FomA, PMSF inhibited this effect of *P. gingivalis* OMVs. (**A**) Morphological changes in *F. nucleatum* were observed by SEM following treatment with *P. gingivalis* OMVs for 6 hours. In addition, the heat‐inactivation group of OMVs treated with heat inactivation at 100°C for 30 minutes, the PMSF group of OMVs treated with PMSF at 37°C for 30 minutes, and the PMSF‐NC group where PMSF was directly added to *F. nucleatum* were also added. Scale bar = 2 μm. (**B**) Bacterial absorbance value assays were used to detect the effect of *P. gingivalis* OMVs on the proliferation of *F. nucleatum*. (**C**) The optical microscope was used to observe the auto‐aggregation morphology of *F. nucleatum* treated with *P. gingivalis* OMVs for 6 hours. Scale bar = 200 μm. (**D**) Representative western blot images of *F. nucleatum* surface adhesion‐related proteins FadA and FomA showing that treatment with *P. gingivalis* OMVs for 6 hours downregulated FadA and FomA expression. DnaK served as an internal control in whole‐bacteria lysates. The data are presented as the mean ± SD obtained from three independent experiments (*n* = 3). *P < 0.05, **P < 0.01 vs. the control cells (Student's *t* test). PMSF, phenylmethylsulfonyl fluoride; PMSF‐NC, phenylmethylsulfonyl fluoride negative control; SEM, scanning electron microscope

In order to further explore the specific mechanism of *P. gingivalis* OMVs inhibiting the invasion of *F. nucleatum* into HIOECs, we hypothesized that *P. gingivalis* OMVs inhibited the invasion ability of *F. nucleatum* by inhibiting the expression of FadA and FomA. We conducted the following experiments to verify this hypothesis. We first observed and photographed the morphology of *F. nucleatum* alone or in the presence of *P. gingivalis* OMVs by SEM. As shown in the results of Figure 3A, the morphology of *F. nucleatum* in all of the four groups were not different from that of the control group, indicating that *P. gingivalis* OMVs did not cause a change in the morphology of *F. nucleatum*. Next, we use a microplate reader to measure the absorbance values at 600 nm at 0, 2, 4, 6, and 8 hours, respectively, and the growth curves of *F. nucleatum* alone or in the presence of *P. gingivalis* OMVs were drawn. As shown in the results of Figure 3B, the proliferation ability of *F. nucleatum* was not affected in all groups. Subsequently, we observed the effect of *P. gingivalis* OMVs on the auto‐aggregation of *F. nucleatum* through an optical microscope. As shown in Figure 3C, the degree of the auto‐aggregation of *F. nucleatum* in the presence of *P. gingivalis* OMVs was significantly reduced, and the arrangement of bacteria was more dispersed. PMSF can inhibit the effect of *P. gingivalis* OMVs to a certain extent and reduce the degree of dispersion of *F. nucleatum*. Finally, the effect of *P. gingivalis* OMVs on the expression of FadA and FomA in *F. nucleatum* was detected with western blotting. As shown in Figure 3D, *P. gingivalis* OMVs downregulated the expression of FadA and FomA proteins, whereas PMSF can reduce the effect of *P. gingivalis* OMVs. There was no difference in the PMSF‐NC group, which ruled out the effect of PMSF on the two proteins of *F. nucleatum*.

## 4 DISCUSSION

Periodontitis is induced by the destruction of the homeostasis between microorganisms, and the interaction between different periodontal pathogens can lead to synergistic pathogenicity.^15^ As the two most common pathogens in periodontitis, the interaction between *P. gingivalis* and *F. nucleatum* is worthy of discussion.

Oral epithelial cells are the first natural barrier of periodontal tissue, and the invasion of periodontal pathogens into oral epithelial cells is a key step in their pathogenesis.^32^ Studies have confirmed that *P. gingivalis* and *F. nucleatum* utilize different invasion strategies.^33^ *P. gingivalis* utilizes the endocytic pathway and lipid rafts to invade host cells.^34^, ^35^ *F. nucleatum* invades host cells through a “zipper” mechanism that relies on a large number of adhesins.^36^

There have been some explorations about the interaction between *P. gingivalis* and *F. nucleatum*. Experiments have confirmed that mixed infection of *P. gingivalis* and *F. nucleatum* could aggravate abscess formation^37^ and alveolar bone loss^38^ in mice with experimental periodontitis. In vitro experiments, mixed infection significantly increased the invasion of *P. gingivalis* into gingival epithelial cells.^33^, ^39^ In addition, it was found that mixed infection increased the invasion of *F. nucleatum* into keratinocytes derived from mouse palatal tissues,^40^ but performed a suppressive effect in the invasion of *F. nucleatum* into human gingival epithelial cells^16^. The contradictory results may be because of differences between bacteria strains and cell types. In the present study, we confirmed that *P. gingivalis* inhibited the invasion of *F. nucleatum* into oral epithelial cells, but *F. nucleatum* promoted the invasion of *P. gingivalis*, which were consistent with previous results.^16^, ^33^, ^39^ *P. gingivalis* can internalize into host cells and make itself long‐term survival^13^, whereas the survival time of *F. nucleatum* is shorter.^6^ The reduction of the invasion of *F. nucleatum* might prevent it from being degraded by host cells, whereas *F. nucleatum* could exert virulence through the paracellular pathway and cooperate with *P. gingivalis* to create a local environment and promote the infection to penetrate deep into the tissues^16^. But they only used a mixed infection method of *P. gingivalis* and *F. nucleatum*, and could not clarify the interaction between the two bacteria and the specific mechanism of their effects.

*P. gingivalis* OMVs could represent the parent bacteria to interact with other bacteria. For example, *P. gingivalis* OMVs enhanced adhesion and invasion of *Tannerella forsythia* to epithelial cells,^25^ and also inhibited and dispersed competitive biofilms in a gingipains dependent manner.^20^ In our research, we extracted *P. gingivalis* OMVs successfully and revealed that *P. gingivalis* OMVs inhibited the invasion of *F. nucleatum* into oral epithelial cells. This trend was consistent with the results shown in previous mixed infection experiments. It indicated that *P. gingivalis* could reduce the invasion ability of *F. nucleatum* through certain toxic components in OMVs, so that *P. gingivalis* itself had an advantage when invading into oral epithelial cells.

**FIGURE 4:**
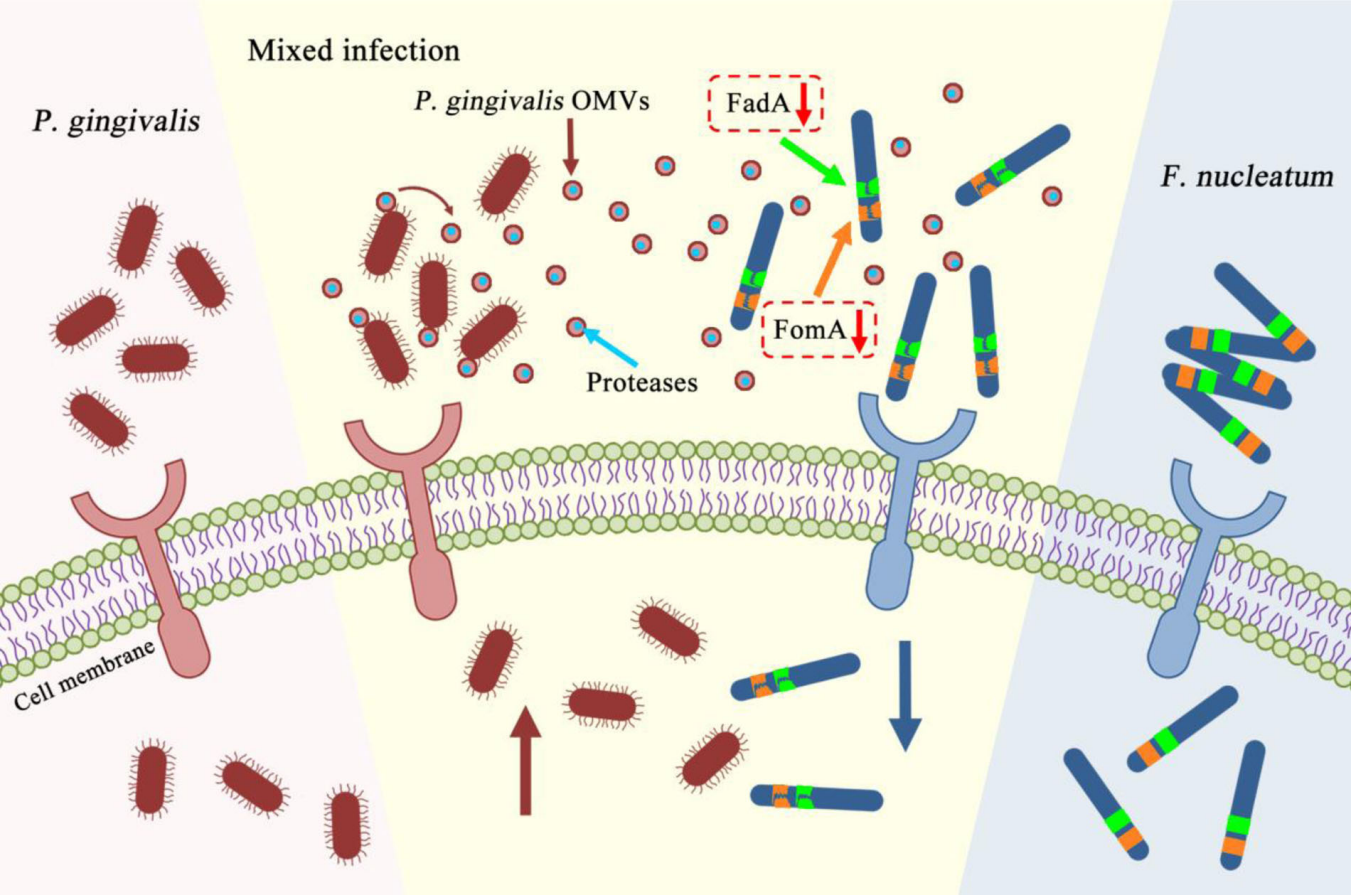
A schematic diagram of how *P. gingivalis* OMVs affect the invasion ability of *F. nucleatum*. In the mixed infection model, *P. gingivalis* inhibited the invasion ability of *F. nucleatum*, whereas *F. nucleatum* promoted the invasion ability of *P. gingivalis* on the contrary. After the treatment of *P. gingivalis* OMVs, the auto‐aggregation of *F. nucleatum* was decreased, the adhesion ability of *F. nucleatum* was reduced, and the expression of FadA and FomA were downregulated, so as to inhibit the invasion of *F. nucleatum* into oral epithelial cells. Proteases in *P. gingivalis* OMVs probably play a major role

The ability of bacteria to invade host cells is affected by many factors. In order to explore the mode of action of *P. gingivalis* OMVs on *F. nucleatum*, we separately studied the morphology, proliferation ability, and auto‐aggregation of *F. nucleatum*. According to the pre‐experimental conditions, we found that *P. gingivalis* OMVs did not affect the biological morphology and proliferation of *F. nucleatum*, but decreased the auto‐aggregation of *F. nucleatum* and made the arrangement of bacteria more dispersed. Furthermore, it is well known that the adhesion ability of *F. nucleatum* is closely related to the expression of considerable adhesins on its surface, which mediate the invasion of *F. nucleatum* into host cells and facilitate the spread of bacteria.^41^, ^42^, ^43^ FadA is an adhesin highly conserved among oral fusobacterial species, including intact pre‐FadA and secreted mature FadA.^44^ FadA binds to E‐cadherin on the cell surface^45^ and participates in adhesion and invasion to host cells.^46^ It is not clear whether FadA is related to auto‐aggregation. FomA is a major outer membrane pore protein of *F. nucleatum*.^47^ FomA binds to the Fc fragment of human immunoglobulin G and works in bacterial co‐aggregation and biofilm formation and is beneficial for the invasion of bacteria into host cells.^48^ In our present study, *P. gingivalis* OMVs reduced the expression levels of FadA and FomA of *F. nucleatum*, whereas this effect could be inhibited by PMSF. We linked this discovery to previous experiments and suggested that *P. gingivalis* OMVs degraded FadA and FomA through protease components, and further inhibited the invasion of *F. nucleatum* into oral epithelial cells (Figure 4).

Proteins contained in *P. gingivalis* OMVs mainly include gingipains,^19^ heme‐binding lipoproteins HmuY and IhtB,^49^ etc. Gingipains are a group of proteases, including Kgp and Rgps. Studies have confirmed that gingipains promoted the invasion of *F. nucleatum* into gingival fibroblasts and macrophages, but had an inhibitory effect on the invasion into gingival epithelial cells.^16^ *P. gingivalis* OMVs carry a concentrated virulence factor, which exert a stronger virulence effect than the parent bacteria^50^. Although our experiments did not directly prove that which specific components of *P. gingivalis* OMVs affected FadA and FomA, heat inactivation or protease inhibitor PMSF can cause *P. gingivalis* OMVs to lose its effect on *F. nucleatum*, indicating that gingipains in *P. gingivalis* OMVs may play a major role.

In conclusion, we found that *P. gingivalis* inhibited the expression of adhesion‐related proteins FadA and FomA of *F. nucleatum* through proteases (probably gingipains) in OMVs, thereby inhibiting the invasion of *F. nucleatum* into oral epithelial cells. In this way, *P. gingivalis* may prevent *F. nucleatum* from being intracellular degraded, make it retain morphology and proliferation activity, and promote deeper infection synergistically. This study provides a new evidence for the interaction between periodontal pathogens, deepens our understanding of *P. gingivalis* as a major pathogen in periodontitis, and may provide new ideas for the pathogenesis of periodontitis.

## CONFLICT OF INTEREST

All authors are from the School and Hospital of Stomatology, China Medical University and declare no conflicts of interest.

## Supporting information

Supplementary figure 1: *P. gingivalis* OMVs increased the mRNA expression levels of FadA and FomA of *F. nucleatum*.
Supplementary figure 2: Western blot of FadA and FomA polyclonal antibody performance: It can be seen that the background of the two antibodies is clean, the bands are clear, and the specificity is good.
