# Supplementary material for: Porphyromonas gingivalis outer membrane vesicles inhibit the invasion of Fusobacterium nucleatum into oral epithelial cells by downregulating FadA and FomA
Source: J Periodontol. 2021 Oct 5;93(4):515–25. doi: 10.1002/JPER.21-0144 (PMC9415117; doi:10.1002/JPER.21-0144)
Supplement: Supplementary file 1 — Supplementary figure 1: P. gingivalis OMVs increased the mRNA expression levels of FadA and FomA of F. nucleatum. Supplementary figure 2: Western blot of FadA and FomA polyclonal antibody performance: It can be seen that the background of the two antibodies is clean, the bands are clear, and the specificity is good. [file JPER-93-515-s001.docx]

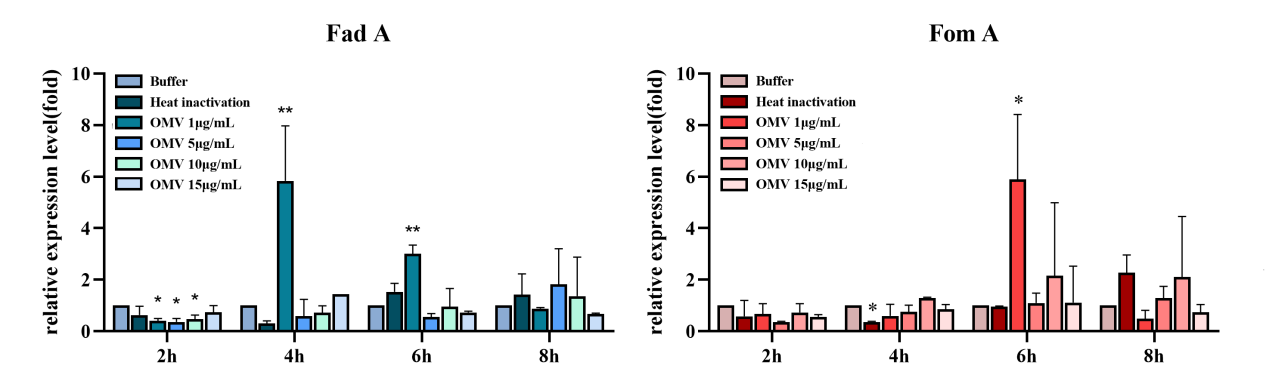
**Supplementary figure 1: *P. gingivalis* OMVs increased the mRNA expression levels of FadA and FomA of *F. nucleatum*. Q-PCR results showed that when the concentration of *P. gingivalis* OMVs is 1μg/mL, FadA increased significantly at 4h, while FadA and FomA both increased at 6h. Therefore, we chose 1μg/mL and 6h as the condition of treatment for this experiment.**


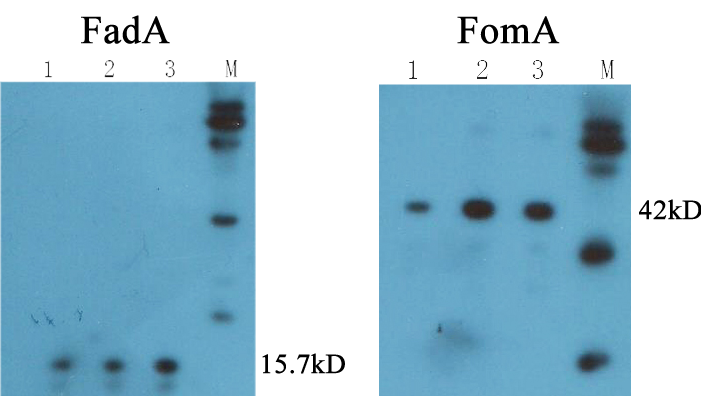


**Supplementary figure 2: Western blot of FadA and FomA polyclonal antibody performance: It can be seen that the background of the two antibodies is clean, the bands are clear, and the specificity is good.**
